# Supplementary material for: The interactome of intact mitochondria by cross-linking mass spectrometry provides evidence for coexisting respiratory supercomplexes
Source: Mol Cell Proteomics. 2017 Dec 8;17(2):216–32. doi: 10.1074/mcp.RA117.000470 (PMC5795388; doi:10.1074/mcp.RA117.000470)
Supplement: Supplemental Data [file supp_RA117.000470_133922_0_supp_23978_fzffwf.zip › spectra_annotation/mito_DR_spectra_annotation/59-1-7-1-6-1.pdf]

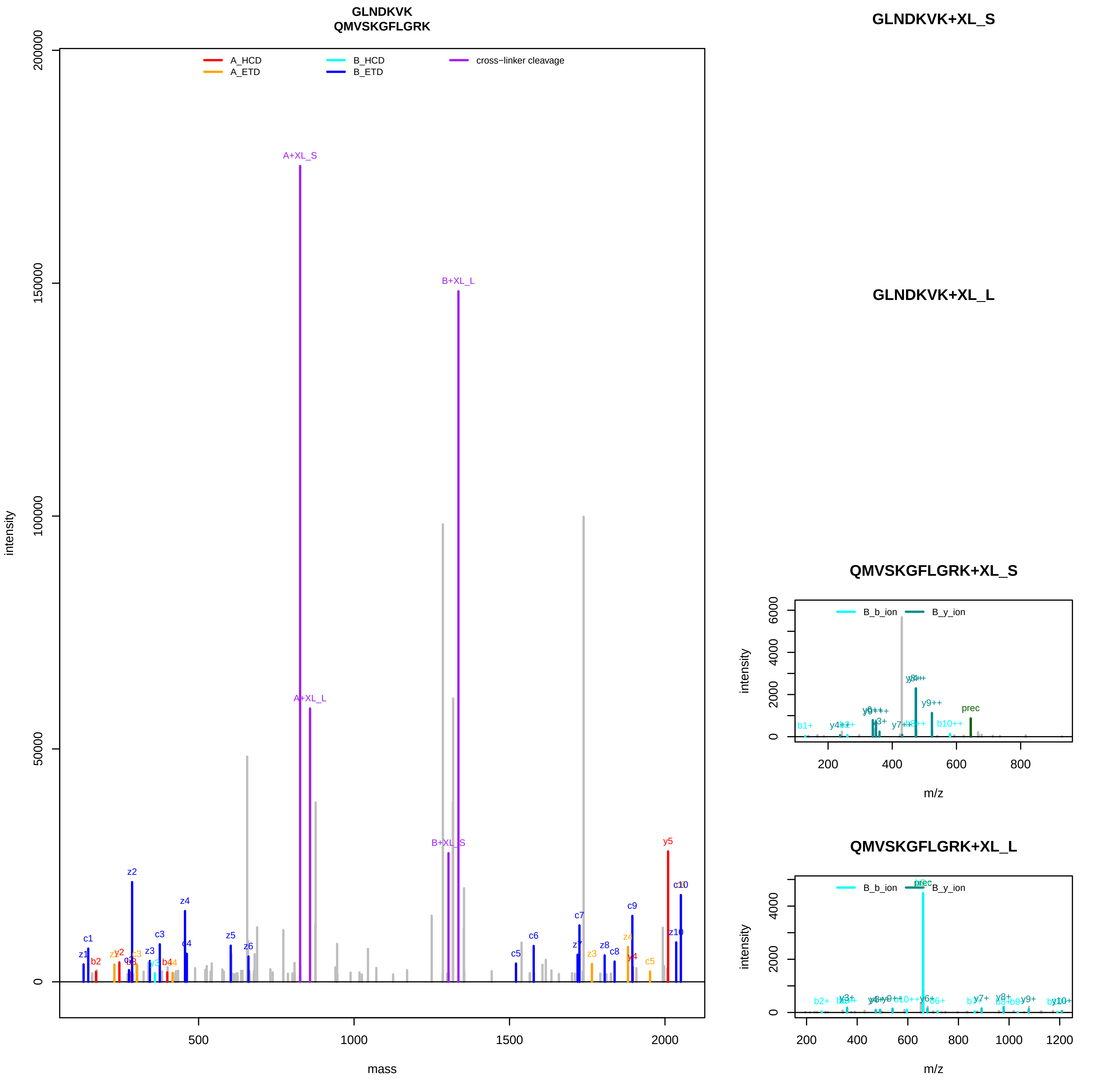
GLNDKVK
QMVSKGFLGRK
A_HCD
A_ETD
B_HCD
B_ETD
cross−linker cleavage
A+XL_S
B+XL_L
A+XL_L
B+XL_S
y5
c10
z10
c9
c7
c6
c5
z2
z4
c3
c4
z5
z6
c1
z1
b2
intensity
mass
0
50000
100000
150000
200000
500
1000
1500
2000
GLNDKVK+XL_S
GLNDKVK+XL_L
QMVSKGFLGRK+XL_S
B_b_ion
B_y_ion
prec
intensity
m/z
0
2000
4000
6000
200
400
600
800
QMVSKGFLGRK+XL_L
B_b_ion
B_y_ion
prec
intensity
m/z
0
2000
4000
200
400
600
800
1000
1200
